# Impact of Normoglycemia in Reducing Microvascular Complications in Patients with Type 2 Diabetes: A Follow-Up Study

**DOI:** 10.3389/fendo.2018.00052

**Published:** 2018-03-01

**Authors:** Fukashi Ishibashi, Mitra Tavakoli

**Affiliations:** ^1^Ishibashi Clinic, Hiroshima, Japan; ^2^University of Exeter Medical School, Exeter, United Kingdom

**Keywords:** microvascular complications, neuropathy outcome, corneal nerve fibers, glycemic control, near-normoglycemia, type 2 diabetes, treatment strategy

## Abstract

**Aims:**

Hyperglycemia is associated with an increased risk of microvascular complications in patients with type 2 diabetes. The aim of the present study was to investigate whether the reduction of the levels of HbA1c by tight glycemic control (GC) decreases the rate of microvascular complications and improves the neurological measures in patients with type 2 diabetes.

**Methods:**

Detailed clinical and neurological examinations including corneal confocal microscopy (CCM) were performed in 141 Japanese patients with type 2 diabetes and 60 age-matched control subjects at baseline and follow-up with GC for 4 years. Patients were stratified according to the mean HbA1c level during follow-up into good (HbA1c < 53.0 mmol/mol, mean; 47.5 mmol/mol), fair (53.0 mmol/mol ≤HbA1c < 58.5 mmol/mol, mean; 55.6 mmol/mol), and poor (HbA1c ≥ 58.5 mmol/mol, mean; 68.9 mmol/mol) GC groups with similar HbA1c levels at baseline (84.5–88.2 mmol/mol).

**Results:**

At baseline, CCM revealed significant nerve fiber damage in all patients compared to that in controls. The interval changes in most corneal nerve fiber (CNF) parameters and neurophysiological functions were significantly related with the mean HbA1c levels during follow-up. Interestingly, the baseline HbA1c level did not impact on neurological functions at follow-up. Interval changes in neuropathy outcomes were associated with mean clinical factors during follow-up and hypoglycemic strategies. Good GC improved all nerve functions, including CNF branch density and bead, but not the length and main fiber density. Fair GC deteriorated some nerve functions. Poor GC compromised all neuropathy outcomes. Irrespective of GC levels, retinopathy increased after follow-up period, while nephropathy decreased.

**Conclusion:**

This study showed that tight GC was beneficial just for nephropathy among microvascular complications. Despite strict GC, the retinopathy progressed in patients with type 2 diabetes. Glucose control did not improve neurophysiological and corneal nerve measurements unless near-normoglycemia was reached.

## Introduction

Hyperglycemia is associated with an increased risk of microvascular complications in patients with type 2 diabetes mellitus (T2DM). Several large clinical trials concluded that intensive glycemic control (GC) in type 2 diabetes is associated with a reduction in microvascular complications, in particular nephropathy and vascular outcomes (1–3).

It has been shown that intensive GC has an equivocal efficacy for diabetic peripheral neuropathy (DPN) in T2DM (4). The earliest nerve fibers to undergo damage and subsequent repair are small nerve fibers (SNFs) (5, 6). Therefore, the functional and morphological measures for SNF neuropathy are essential for estimating the efficacy of intervention for DPN.

Small nerve fibers can be assessed objectively by quantifying intraepidermal nerve fiber density in skin biopsies; however, this is an invasive procedure that requires expert laboratory assessment and has considerable variability even among control (7). The use of corneal confocal microscopy (CCM) for rapid, noninvasive clinical assessment of corneal nerve fibers (CNFs) has grown significantly in recent years (8). It has proven to be particularly useful as a diagnostic marker for the detection of diabetic neuropathy (9–11) and a range of other peripheral neuropathies (12–14).

Due to potential severe hypoglycemia (3), euglycemia using insulin-providing strategy in T2DM patients may be difficult. The benefit of strict GC on neurophysiological functions, CNF parameters, and other microangiopathies markers in patients with poorly controlled type 2 diabetes is unclear and needs to be investigated.

The aim of the present study is to establish whether the tight control of HbA1c improves the CNF measures and neurophysiological functions along with the occurrence of nephropathy and retinopathy in patients with poorly controlled type 2 diabetes by strict GC for 4 years.

## Subjects and Methods

### Subjects

At the baseline, 141 Japanese patients with poorly controlled type 2 diabetes (HbA1c > 58.5 mmol/mol) who underwent GC for 3–5 years and 60 healthy control subjects without diabetes (HbA1c < 38.8 mmol/mol) were enrolled between June 2011 and July 2012. The patients were followed up for 4 years in average (March 2015 to November 2016) at Ishibashi Clinic, Hiroshima, Japan. The patients with diabetes were divided into the following three subgroups according to baseline HbA1c levels: subgroup 1; 58.5 mmol/mol < HbA1c ≤ 74.9 mmol/mol, subgroup 2; 74.9 mmol/mol < HbA1c ≤ 91.3 mmol/mol, and subgroup 3; HbA1c > 91.3 mmol/mol, and also according to the mean HbA1c level during follow-up [good (HbA1c < 53.0 mmol/mol), fair (53.0 mmol/mol ≤ HbA1c < 58.5 mmol/mol), and poor (HbA1c ≥ 58.5 mmol/mol) control subgroups]. The patients assigned to the insulin-sensitizing or insulin-providing strategy were treated with biguanides or pioglitazone, or with sulfonylureas or insulin, respectively (15).

The HbA1c for patients was measured monthly during the follow-up period. And hence, the mean HbA1c levels during follow-up are representative, and the linear regression provided changes in neuropathy outcomes by reduction in the mean HbA1c level per year of follow-up.

The exclusion criteria were any kind of other neuropathy apart from diabetes, vitamin B deficiency, and severe nonproliferative or proliferative diabetic retinopathy defined by Early Treatment Diabetic Retinopathy Study (ETDRS) (16), any type of corneal disease and history of refractive surgery, using contact lenses and significant media opacity at baseline and end point. Written informed consent was obtained from all subjects based on the Declaration of Helsinki. The ethics committee of Ishibashi Clinic approved the protocol of this study.

### Corneal Confocal Microscopy

All study subjects were examined using a Heidelberg Retina Tomograph (III) *in vivo* CCM (Rostock Corneal module, Heidelberg Engineering, Heidelberg, Germany) (17). Patients with T2DM underwent CCM examination at baseline and end point. Control subjects underwent CCM examination once at the start of the study. A minimum of six high-quality images per subject from the sub-basal nerve plexus and Bowman’s layer from the center of cornea was selected and quantified for the following CNF parameters: (1) corneal nerve fiber density (CNFD), the total number of major nerve fibers/mm^2^ of corneal tissue; (2) corneal nerve fiber length (CNFL), the total length of all nerve fibers (mm/mm^2^); (3) corneal nerve branch density (CNBD), the number of branches emanating from all major nerve trunks/mm^2^; (4) beading frequency (BF) (/0.1 mm); and (5) bead size (BS) (μm^2^) determined after enlarging five times and smoothing the original image of CCM using S-Spline Max algorithm (PhotoZoomPro4, Gungle Inc., Tokyo, Japan). The pixel numbers of 120 beads were counted using Photoshop Elements 8.0 (Adobe Systems Inc., San Jose, CA, USA) and averaged (18). Except for BS, all measurements were performed using ImageJ (Texelcraft, Tokyo, Japan).

### Assessment of Neuropathy and Neurophysiological Examinations

All healthy control subjects and patients with T2DM underwent detailed neurophysiological examinations at baseline and final follow-up visits. The neurological deficits were assessed using the modified neuropathy disability score (NDS) (19), which includes evaluations of vibration perception, pin prick, temperature perception, and ankle reflexes. The classification for the evaluation of neuropathy was based on the Toronto consensus (20) which considered combination of symptoms (numbness or reduced ability to feel pain or temperature changes, a tingling or a burning sensation, sharp pains, increased sensitivity to touch, and muscle weakness), signs (a symmetric decrease in distal sensation and decreased or the absence of ankle reflexes), and electrophysiological tests. Hence, the patients with NDS >2 and sensory nerve conduction velocity (SCV) of sural nerve (SN) <42 m/s were labeled with neuropathy based on the Toronto criteria.

Electrophysiology and nerve conduction velocity (NCV) studies were performed using an electromyography instrument (Neuropak S1, NIHON KOHDEN, Tokyo, Japan). The motor nerve conduction velocity (MCV) of median nerve (MN) and sensory (SCV, ulnar, and SN) NCVs and their action potential amplitudes were determined. Skin temperature was maintained above 32°C.

The vibration perception threshold (VPT) was measured at the left medial malleolus using a biothesiometer (Biomedical Instruments, Newbury, OH, USA). The warm and cold perception thresholds (PTs) at the dorsum of the foot were determined using a thermal stimulator (Intercross-200, Intercross Co., Tokyo, Japan). To assess cardiac autonomic neuropathy (CAN), the coefficient of variation of R–R intervals (CV_R–R_) was calculated from the R–R intervals of 200 samples on electrocardiogram.

### Clinical and Laboratory Data

The BMI, blood pressure, and HbA1c levels were measured monthly during the terms of study (48 months in average). The measured HbA1c levels were converted to National Glycohemoglobin Standardization Program units (21) and subsequently to International Federation of Clinical Chemistry values. The serum lipid levels [low-density lipoprotein (LDL) cholesterol (C), high-density lipoprotein (HDL)-C, and triglycerides], and urinary creatinine and albumin levels were assessed every 3 months. The albumin-to-creatinine ratio (ACR) >30 mg/gCr twice a year was labeled as nephropathy (22).

At baseline and end point, bilateral retinal fundus images were captured (the field of assessment: 45°) and graded according to the ETDRS scale: no apparent retinopathy, 0; mild nonproliferative diabetic retinopathy, 1; moderate nonproliferative diabetic retinopathy, 2 (16).

### Statistical Analysis

Statistical analyses were performed using SPSS ver. 19 (SPSS, Chicago, IL, USA). A *p*-value of <0.05 was considered to be statistically significant.

The *post hoc* analysis of sample power revealed that by using a one-sided ANOVA (significance of 0.05) and Kruskal–Wallis test for CNF measures and neurophysiological tests, the present study population provided statistical power ranging from 0.71 to 0.99. All values are presented as mean ± standard error of the mean (SEM). All data sets were tested for normality using the Shapiro–Wilk test. For normally distributed variables, comparisons between controls and patients with diabetes (baseline, end point, and mean value during follow-up) or between controls and each diabetic subgroups divided by the baseline or the mean HbA1c levels were performed using one-way ANOVA for continuous variables and the χ^2^-test for categorical variables followed by Bonferroni correction. For non-normally distributed variables, the Kruskal–Wallis test was applied followed by the Mann–Whitney *U*-test and Bonferroni correction for continuous variables and the McNemar test for categorical variables. The differences between baseline and end point were assessed using the paired *t*-test and Wilcoxon’s signed rank test for normally and non-normally distributed continuous variables, respectively. Correlations between the neuropathy outcome measures and the mean clinical factors during follow-up or treatment strategies were assessed using Spearman’s rank correlation coefficient or multiple regression analysis. The sensitivity, specificity, positive-predictive value (PPV), and negative-predictive value (NPV) of CNF measures and neurophysiological tests in differentiating between control subjects and patients with or without neuropathy were assessed at baseline and end point using receiver-operating characteristic analysis.

## Results

### Clinical, Neurophysiological, and CNF Measure Data of Controls and Patients with Type 2 Diabetes and Their Subgroups Based on Baseline HbA1c Levels

#### At Baseline

**Figure 1:**
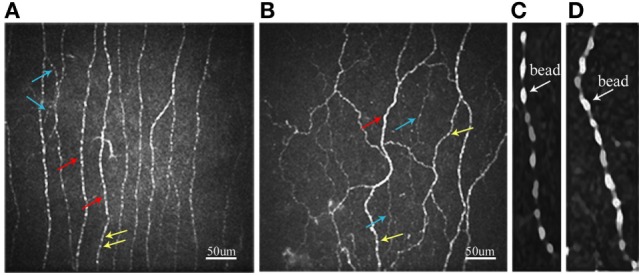
Representative confocal microscopic images of the corneal sub-basal nerve plexus in control subject **(A)** and patient with type 2 diabetes **(B)**. Red arrows, main nerve fiber; blue arrows, branch; yellow arrows, bead. Beading image after enlarging five times with smoothing by the S-Spline Max algorithm in control subject **(C)** and patient with type 2 diabetes **(D)**.

The HbA1c levels were clearly different in three subgroups based on baseline results. The retinopathy was presented in 21.3% of total patients and between 18.2 and 23.4% in subgroups based on ETDRS scale (16). A total of 37.6% patients and 34.1–42.6% of subgroups had nephropathy based on the nephropathy definition by American Diabetes Association (22). The neuropathy was found to be 17.7% in total patients and 14.0–21.3% in subgroups based on Toronto criteria (20). Most neurophysiological tests (NDS, MCV and amplitude of MN, SCV and amplitude of ulnar nerve, SN amplitude, VPT, CV_R–R_, warm and cold PTs, and most CNF measures) were impaired in patients or subgroups compared to those in controls (Table 1). Figure 1 shows representative corneal sub-basal nerve plexuses in a control subject (Figure 1A) and a patient with type 2 diabetes (Figure 1B). Compared with the control, the diabetic patient had reduced CNFD and increased tortuosity.

**Table 1:** Clinical characteristics, neurophysiological tests, corneal nerve fiber measures, hypoglycemic strategies, and incidence of microvascular complications in control subjects, total diabetic patients, and their subgroups divided by the baseline HbA1c levels at the baseline and end point.

	Control	T2DM—Total		Patients with type 2 diabetes stratified based on HbA1c level at baseline					
		
	Control subjects	T2DM	T2DM	Subgroup 1		Subgroup 2		Subgroup 3	
				58.5 < HbA1c ≤ 74.9 mmol/mol		74.9 < HbA1c ≤ 91.3 mmol/mol		HbA1c > 91.3 mmol/mol	
					
		Baseline	End point	Baseline	End point	Baseline	End point	Baseline	End point
Number (M/F)	60 (40/20)	141 (98/43)	141 (98/43)	50 (37/13)	50 (37/13)	44 (30/14)	44 (30/14)	47 (31/16)	47 (31/16)
Age (years)	53.1 ± 0.9	53.2 ± 0.7	57.1 ± 0.7	53.8 ± 1.0	57.7 ± 1.0	53.6 ± 1.2	57.7 ± 1.3	52.2 ± 1.5	55.9 ± 1.5
Follow-up period (years)			4.0 ± 0.1		4.0 ± 0.2		4.1 ± 0.2		3.7 ± 0.1
Duration of diabetes (years)		8.9 ± 0.6	12.9 ± 0.7	11.7 ± 1.1	15.7 ± 1.1	9.6 ± 1.2	13.6 ± 1.2	5.2 ± 0.8^*,†^	8.9 ± 0.8^*,†^
Smoking (%)	26.7	31.9	24.8	38.0	30.0	29.5	20.5	27.7	23.4
Alcohol consumption (%)	40.0	36.9	37.6	52.0	50.0	40.9	40.9	17.0	21.3
Body mass index (kg/m^2^)	22.9 ± 0.5	26.3 ± 0.4^‡^	26.4 ± 0.4	26.2 ± 0.6^‡^	26.4 ± 0.7	26.7 ± 0.7^‡^	26.6 ± 0.8	26.0 ± 0.7^‡^	26.2 ± 0.7
Systolic blood pressure (mmHg)	132 ± 1.8	148 ± 1.7^‡^	139 ± 0.8^§^	144 ± 1.9^‡^	138 ± 1.3^||^	150 ± 3.1^‡^	140 ± 1.2^¶^	150 ± 3.7^‡^	139 ± 1.6^¶^
Diastolic blood pressure (mmHg)	77.8 ± 0.9	88.4 ± 0.7^‡^	80.3 ± 0.6^§^	86.7 ± 0.9^‡^	80.2 ± 1.2^§^	89.1 ± 1.2^‡^	80.9 ± 0.8^§^	90.0 ± 1.7^‡^	80.2 ± 1.1^§^
HbA1c (mmol/mol)	36.8 ± 0.4	85.8 ± 1.8^‡^	55.2 ± 0.9^‡,§^	66.4 ± 0.7^‡^	56.3 ± 1.1^‡,§^	82.6 ± 0.8^‡,*^	56.4 ± 2.0^‡,§^	110 ± 2.4^‡,*,†^	53.0 ± 1.7^‡,§^
LDL cholesterol (mmol/L)	3.23 ± 0.09	3.66 ± 0.09^#^	3.33 ± 0.08^§^	3.40 ± 0.14	3.30 ± 0.14	3.78 ± 0.15^#^	3.48 ± 0.12	3.81 ± 0.17	3.21 ± 0.13^§^
HDL cholesterol (mmol/L)	1.74 ± 0.06	1.47 ± 0.04^‡^	1.43 ± 0.04	1.43 ± 0.05**	1.41 ± 0.06	1.44 ± 0.05**	1.44 ± 0.06	1.53 ± 0.07	1.42 ± 0.07^||^
Triglycerides (mmol/L)	1.53 ± 0.14	2.45 ± 0.18^‡^	1.96 ± 0.12^¶^	2.17 ± 0.20^#^	2.17 ± 0.22	2.40 ± 0.30^#^	1.79 ± 0.15	2.79 ± 0.41**	1.89 ± 0.24
eGFR (mL/min)	81.2 ± 1.9	87.7 ± 1.7^#^	73.6 ± 1.5^§^	80.7 ± 2.2	71.1 ± 2.3^§^	87.7 ± 2.8	76.7 ± 2.7^§^	95.0 ± 3.5^**,††^	73.4 ± 2.7^§^
Albumin-creatinine ratio (mg/gCr)	7.9 ± 1.2	94.9 ± 31.2^‡^	55.4 ± 16.6^§^	31.9 ± 5.9^‡^	41.7 ± 19.2	57.4 ± 19.4^‡^	33.2 ± 18.3^¶^	197 ± 90.3^‡^	90.8 ± 41.9^§^
ETDRS retinopathy scale	0	0.40 ± 0.06	0.62 ± 0.08^§^	0.48 ± 0.12	0.54 ± 0.13	0.34 ± 0.10	0.66 ± 0.13^¶^	0.36 ± 0.11	0.66 ± 0.13^¶^

**Neurophysiological tests**									
Neuropathy disability score (0–10)	0	4.2 ± 0.2	4.1 ± 0.2	3.7 ± 0.3	3.7 ± 0.3	4.6 ± 0.3	4.6 ± 0.3	4.4 ± 0.4	4.0 ± 0.4
MCV of median nerve (m/s)	58.1 ± 0.44	52.3 ± 0.41^‡^	52.7 ± 0.42	53.3 ± 0.72^‡^	52.5 ± 0.76	51.5 ± 0.60^‡^	52.4 ± 0.71	52.1 ± 0.75^‡^	53.1 ± 0.74
Amplitude of median nerve (mV)	7.98 ± 0.31	5.91 ± 0.22^‡^	5.76 ± 0.21	6.00 ± 0.38^‡^	5.72 ± 0.37	5.59 ± 0.36^‡^	5.36 ± 0.35	6.12 ± 0.39**	6.19 ± 0.39
SCV of ulnar nerve (m/s)	63.9 ± 0.51	59.0 ± 0.37^‡^	59.0 ± 0.42	60.2 ± 0.59^‡^	59.5 ± 0.64	58.0 ± 0.59^‡^	58.4 ± 0.83	58.5 ± 0.69^‡^	59.2 ± 0.72
Amplitude of ulnar nerve (μV)	30.5 ± 2.0	15.4 ± 0.53^‡^	14.7 ± 0.51^||^	16.4 ± 0.82^‡^	15.9 ± 0.86	15.3 ± 0.99^‡^	14.4 ± 0.91	14.3 ± 0.93^‡^	13.7 ± 0.89
SCV of sural nerve (m/s)	46.9 ± 0.53	46.2 ± 0.39	45.3 ± 0.43^§^	46.4 ± 0.65	45.2 ± 0.68	46.0 ± 0.72	44.8 ± 0.84	46.2 ± 0.67	45.9 ± 0.74
Amplitude of sural nerve (μV)	14.5 ± 0.84	8.90 ± 0.25^‡^	8.26 ± 0.28^§^	10.0 ± 0.39**	9.35 ± 0.48	8.23 ± 0.39^‡^	7.58 ± 0.46	8.33 ± 0.47^‡,‡‡^	7.74 ± 0.48^‡‡^
VPT (μ/120 c/s)	2.06 ± 0.24	3.73 ± 0.16^‡^	3.74 ± 0.19	3.51 ± 0.26^‡^	3.53 ± 0.25	3.97 ± 0.37^‡^	4.11 ± 0.48	3.74 ± 0.20^‡^	3.60 ± 0.24
CV_R–R_ (%)	3.90 ± 0.12	3.24 ± 0.10^‡^	3.04 ± 0.11^¶^	3.28 ± 0.15^#^	2.97 ± 0.16	3.18 ± 0.20^#^	2.79 ± 0.20	3.28 ± 0.18^#^	3.35 ± 0.19
Warm perception threshold (W/m^2^)	−512 ± 11.8	−580 ± 11.0^‡^	−596 ± 12.9	−579 ± 21.3	−597 ± 22.2	−591 ± 18.4^#^	−600 ± 20.3	−571 ± 17.1	−572 ± 19.6
Cold perception threshold (W/m^2^)	478 ± 11.1	536 ± 7.5^‡^	545 ± 8.2^||^	538 ± 13.9^#^	557 ± 15.2	549 ± 13.1^#^	558 ± 14.2	536 ± 7.5	519 ± 12.3

**Corneal nerve fiber measures**									
Density (no/mm^2^)	32.2 ± 0.74	17.6 ± 0.5^‡^	16.8 ± 0.5	18.5 ± 0.87^‡^	17.7 ± 0.79	17.1 ± 0.90^‡^	15.2 ± 0.88	17.1 ± 0.94^‡^	17.4 ± 0.98
Length (mm/mm^2^)	15.4 ± 0.30	9.08 ± 0.23^‡^	8.83 ± 0.22	9.62 ± 0.41^‡^	9.17 ± 0.37	8.79 ± 0.39^‡^	8.34 ± 0.37	8.77 ± 0.40^‡^	8.93 ± 0.42
Branch density (no/mm^2^)	13.2 ± 0.67	12.2 ± 0.40	12.2 ± 0.50	11.7 ± 0.79	9.90 ± 0.73	13.6 ± 0.75^††^	13.6 ± 0.83^††^	11.6 ± 0.75	13.2 ± 0.90^‡‡^
Beading frequency (no/0.1 mm)	23.1 ± 0.30	19.7 ± 0.20^‡^	19.6 ± 0.20	20.0 ± 0.32^‡^	19.5 ± 0.26	19.4 ± 0.43^‡^	19.0 ± 0.33	19.7 ± 0.38^‡^	20.3 ± 0.33
Bead size (μm^2^)	7.78 ± 0.14	10.4 ± 0.07^‡^	10.2 ± 0.10	9.94 ± 0.11^‡^	10.2 ± 0.15	10.7 ± 0.08^‡,*^	10.4 ± 0.18	10.5 ± 0.11^‡,*^	10.1 ± 0.17

**Hypoglycemic strategies**									
Insulin providing (%)		55.3	66.7	66.0	76.0	56.8	70.5	42.6	53.2^‡‡^
Insulin sensitizing (%)		44.0	75.2^§^	66.0	80.0	43.2^‡‡^	72.7^¶^	21.3*	72.3^§^
DPP-4 inhibitor (%)		19.1	60.3^§^	28.0	72.0^§^	15.9	56.8^§^	12.8	51.1^§^

**Presence of microvascular complications**									
Presence of nephropathy (%)	0	37.6	22.0^§^	36.0	24.0	34.1	15.9^||^	42.6	25.5^||^
Presence of retinopathy (%)	0	21.3	35.5^§^	22.0	28.0	18.2	40.9^¶^	23.4	38.3^||^
Presence of neuropathy (%)	0	17.7	21.3	14.0	16.0	18.2	31.8	21.3	17.0

*Data are the mean ± standard error of the mean in the control subjects, at baseline, and end point in total patients with type 2 diabetes and their subgroups divided by baseline HbA1c levels*.

*p < 0.001 compared with subgroup 1, ^†^p < 0.01 compared with subgroup 2, ^‡^p < 0.001 compared with control subjects, ^§^p < 0.001 compared with baseline, ^||^p < 0.05 compared with baseline, ^¶^p < 0.01 compared with baseline, ^#^p < 0.05 compared with control subjects.

**p < 0.01 compared with control subjects, ^††^p < 0.01 compared with subgroup 1, ^‡‡^p < 0.05 compared with subgroup 1.

*CV_R–R_, coefficient of variation of R–R interval; DPP-4, dipeptidyl peptidase-4; eGFR, estimated glomerular filtration rate; ETDRS, Early Treatment Diabetic Retinopathy Study; HDL, high-density lipoprotein; LDL, low-density lipoprotein; MCV, motor nerve conduction velocity; SCV, sensory nerve conduction velocity; VPT, vibration perception threshold*.

The BS in patient with type 2 diabetes (Figure 1D) is larger than that in control subject (Figure 1C).

#### At End Point

In all patients, the HbA1c level improved by average 30.6 mmol/mol by tight GC. HbA1c levels in all subgroups were similar (53.0–56.4 mmol/mol). In all patients and in particular subgroups 2 and 3, the retinopathy significantly increased and progressed; however, the nephropathy significantly decreased. The incidence of neuropathy did not significantly change in all subgroups. Overall, the GC was not beneficial for improving the neurophysiological functions and CNF parameters, and some neurophysiological functions rather deteriorated after GC (Table 1). Because GC did not improve the functions of large fibers assessed by NCVs and VPT, or neither of SNFs (CNF and temperature perceptions) in all patients, the differential impact by GC on large and SNFs was not obtained (Table 1).

After follow-up among the patient subgroups, despite strict GC and improvement in mean HbA1c by around 30.6 mmol/mol, the cumulative incidence of neuropathy increased from 17.7% at baseline to 21.3% (*p* = 0.383) and retinopathy increased from 21.3 to 35.5% (*p* < 0.001); however, the cumulative incidence of nephropathy reduced from 37.6 to 22.0% (*p* < 0.001) (Table 1). Neuropathy decreased insignificantly in subgroup 3 (those with the poorest GC at baseline which suffered from severe neuropathy).

### Clinical, Neurophysiological, and CNF Measure Data in the Subgroups of Patients with Type 2 Diabetes Stratified by the Mean HbA1c Levels during Follow-Up

Because the baseline HbA1c levels did not impact on neuropathy outcomes, we stratified patients by the mean HbA1c levels during follow-up.

#### At Baseline

The duration of diabetes in good GC subgroup was significantly shorter than others. The percentage of each original subgroup stratified by baseline HbA1c levels in good, fair, and poor control patients was not significantly different. The percentage of subgroup 3 in good control patients seemed to be larger than those in other two groups. The HbA1c levels in all subgroups were similar. Diabetic retinopathy was found more under fair and poor GC than under good GC. There was no difference in the prevalence of nephropathy and neuropathy, and the results of all neuropathy outcome measures between subgroups. Insulin-providing and insulin-sensitizing agents were more frequently prescribed under fair and poor GC than under good GC (Table 2). There was no difference in smoking habit (28.6–34.1%) and alcohol intake (31.8–39.6%) in all subgroups.

**Table 2:** Clinical characteristics, neurophysiological tests, corneal nerve fiber measures, and hypoglycemic strategies at baseline, at end point, and mean values in the diabetic subgroups divided by mean HbA1c levels during follow-up period.

	Grouped based on mean HbA1c level during follow-up period								
	Good control			Fair control			Poor control		
	HbA1c < 53.0 mmol/mol			53.0 ≤ HbA1c < 58.5 mmol/mol			HbA1c ≥ 58.5 mmol/mol		
	Baseline	End point	Mean	Baseline	End point	Mean	Baseline	End point	Mean
Number (M/F)	48 (33/15)	48 (33/15)		49 (35/14)	49 (35/14)		44 (30/14)	44 (30/14)	
Subgroups 1/2/3 (%)	27.1/29.2/43.7			42.8/28.6/28.6			36.4/36.4/27.2		
Age (years)	51.9 ± 1.4	55.7 ± 1.4	53.8 ± 1.4	56.1 ± 1.1*	60.3 ± 1.1*	58.2 ± 1.1*	51.3 ± 1.2^†^	55.1 ± 1.2^‡^	53.4 ± 1.3^†^
Follow-up period (years)		3.8 ± 0.1			4.2 ± 0.2			4.0 ± 0.1	
Duration of diabetes (years)	4.2 ± 0.6	7.9 ± 0.6	6.1 ± 0.6	9.6 ± 1.2*	13.8 ± 1.2*	11.7 ± 1.2*	13.1 ± 1.1^§^	17.0 ± 1.1^§^	15.1 ± 1.1^§^
Body mass index (kg/m^2^)	26.3 ± 0.7	25.3 ± 0.6^||^	24.9 ± 0.6	26.2 ± 0.7	26.6 ± 0.8	26.1 ± 0.8	26.3 ± 0.7	27.3 ± 0.8^||^	26.9 ± 0.7
Systolic blood pressure (mmHg)	148 ± 3.2	137 ± 1.1^¶^	137 ± 1.1	151 ± 2.9	140 ± 1.4^||^	140 ± 1.1	143 ± 2.5^†^	139 ± 1.6	141 ± 1.1
Diastolic blood pressure (mmHg)	89.4 ± 1.4	79.8 ± 1.0^#^	81.7 ± 0.9	89.6 ± 1.4	80.0 ± 1.1^#^	82.1 ± 0.8	85.9 ± 1.0	81.6 ± 0.9^||^	83.5 ± 0.6
HbA1c (mmol/mol)	88.2 ± 2.7	47.4 ± 0.7^#^	47.5 ± 0.5	84.5 ± 3.2	53.9 ± 0.5^#,§^	55.6 ± 0.2^§^	85.2 ± 3.3	64.4 ± 2.2^#,§,‡^	68.9 ± 1.7^§,**^
Decrease in HbA1c from baseline (mmol/mol)		−40.8 ± 3.0			−30.6 ± 3.4*			−20.8 ± 3.9^§^	
LDL cholesterol (mmol/L)	3.81 ± 0.15	3.28 ± 0.12^#^	3.27 ± 0.10	3.68 ± 0.17	3.16 ± 0.14^||^	3.19 ± 0.11	3.44 ± 0.14	3.56 ± 0.13	3.50 ± 0.11
HDL cholesterol (mmol/L)	1.37 ± 0.06	1.40 ± 0.08	1.40 ± 0.05	1.49 ± 0.06	1.43 ± 0.06	1.45 ± 0.06	1.55 ± 0.07	1.44 ± 0.06	1.47 ± 0.06
Triglycerides (mmol/L)	2.43 ± 0.25	2.00 ± 0.24	1.81 ± 0.13	2.42 ± 0.25	1.89 ± 0.20	1.76 ± 0.12	2.50 ± 0.43	1.98 ± 0.19	1.96 ± 0.23
Presence of nephropathy (%)	27.1	10.4^¶^		38.8	26.5		47.7	29.5	
Presence of retinopathy (%)	4.2	10.4		24.5*	34.7		36.4^§^	63.6^#,§^	
Presence of neuropathy (%)	18.8	6.3^¶^		16.3	18.4		18.2	40.9^||,§,†^	

**Neurophysiological tests**									
MCV of median nerve (m/s)	53.3 ± 0.51	55.9 ± 0.51^#^		51.6 ± 0.79	51.4 ± 0.78^§^		52.1 ± 0.78	50.5 ± 0.62^§,||^	
Amplitude of median nerve (mV)	5.92 ± 0.38	6.41 ± 0.37^¶^		5.95 ± 0.35	5.68 ± 0.37		5.86 ± 0.41	5.14 ± 0.38^*,||^	
SCV of ulnar nerve (m/s)	59.5 ± 0.55	61.7 ± 0.60^#^		58.5 ± 0.64	58.4 ± 0.66^††^		59.0 ± 0.72	56.8 ± 0.74^§,#^	
Amplitude of ulnar nerve (μV)	16.5 ± 0.98	17.9 ± 0.91^#^		14.6 ± 0.90	13.9 ± 0.81*		15.0 ± 0.84	12.1 ± 0.74^§,#^	
SCV of sural nerve (m/s)	46.3 ± 0.74	47.6 ± 0.72^#^		46.4 ± 0.70	45.0 ± 0.74^#^		45.9 ± 0.56	43.1 ± 0.63^#,§^	
Amplitude of sural nerve (μV)	8.82 ± 0.39	9.43 ± 0.44^||^		9.04 ± 0.48	8.40 ± 0.51^#^		8.82 ± 0.44	6.82 ± 0.44^#,§^	
VPT (μ/120 c/s)	3.60 ± 0.24	2.85 ± 0.22^#^		3.80 ± 0.24	3.82 ± 0.25*		3.80 ± 0.36	4.62 ± 0.46^#,§^	
CV _R–R_ (%)	3.24 ± 0.16	3.53 ± 0.18^#^		3.23 ± 0.21	3.04 ± 0.21^||,††^		3.27 ± 0.15	2.52 ± 0.13^#,§^	
Warm perception threshold (W/m^2^)	−564 ± 14.8	−520 ± 13.2^#^		−593 ± 23.0	−618 ± 24.0^#,††^		−582 ± 18.3	−633 ± 19.7^#,§^	
Cold perception threshold (W/m^2^)	532 ± 12.8	497 ± 13.3^#^		544 ± 14.1	557 ± 13.4^||,††^		532 ± 11.7	583 ± 12.9^#,§^	

**Corneal nerve fiber measures**									
Density (no/mm^2^)	17.9 ± 0.9	18.2 ± 0.7		18.3 ± 1.0	17.6 ± 0.9		16.4 ± 0.8	14.5 ± 1.0^††,||^	
Length (mm/mm^2^)	9.01 ± 0.34	9.44 ± 0.33		9.31 ± 0.46	9.20 ± 0.40		8.90 ± 0.39	7.75 ± 0.40^††,#^	
Branch density (no/mm^2^)	11.4 ± 0.6	13.6 ± 0.9^#^		12.0 ± 0.9	12.3 ± 0.9		13.4 ± 0.8	10.5 ± 0.7^#^	
Beading frequency (no/0.1 mm)	19.6 ± 0.3	20.9 ± 0.3^||^		19.8 ± 0.4	19.7 ± 0.2		19.7 ± 0.3	18.1 ± 0.3^#,§,‡^	
Bead size (μm^2^)	10.4 ± 0.1	9.4 ± 0.1^#^		10.5 ± 0.1	10.2 ± 0.1^¶^		10.3 ± 0.1	11.2 ± 0.2^#,§,†^	

**Hypoglycemic strategies**									
Insulin providing (%)	22.9	8.3		63.3^§^	93.9^§,#^		81.8^§^	100^§,||^	
Insulin sensitizing (%)	18.8	77.1^#^		49.0^§^	71.4^¶^		65.9^§^	77.3	
DPP-4 inhibitor (%)	8.3	39.6^#^		26.5	65.3^*,#^		22.7	77.3^§,#^	

*Data are the mean ± standard error of the mean at baseline, at end point, and mean values during follow-up in the subgroups of patients with type 2 diabetes divided by the mean HbA1c levels during follow-up period*.

*p < 0.05 compared with good control subgroup, ^†^p < 0.05 compared with fair control subgroup, ^‡^p < 0.01 compared with fair control subgroup, ^§^p < 0.001 compared with good control subgroup, ^||^p < 0.01 compared with baseline, ^¶^p < 0.05 compared with baseline, ^#^p < 0.001 compared with baseline, **p < 0.001 compared with fair control subgroup, ^††^p < 0.01 compared with good control subgroup.

*CV_R–R_, coefficient of variation of R–R interval; DPP-4, dipeptidyl peptidase-4; HDL, high-density lipoprotein; LDL, low-density lipoprotein; MCV, motor nerve conduction velocity; SCV, sensory nerve conduction velocity; VPT, vibration perception threshold*.

#### At End Point and Mean Levels

**Figure 2:**
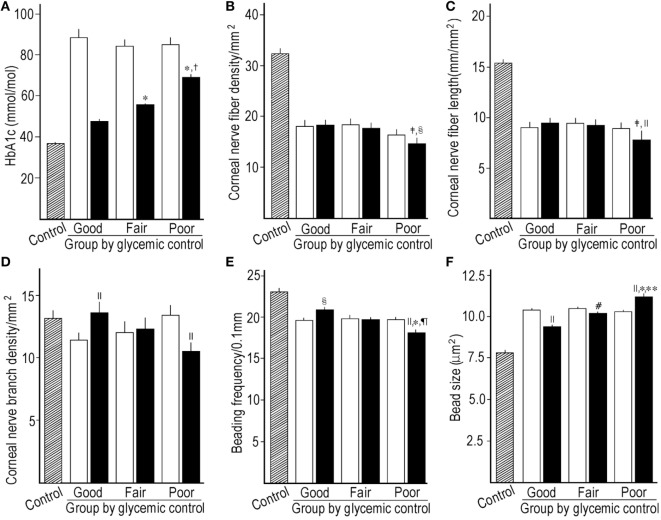
Comparison of HbA1c between control subjects, baseline, and mean levels during follow-up among subgroups stratified by the mean HbA1c levels during follow-up **(A)**, corneal nerve fiber density (CNFD) **(B)**, corneal nerve fiber length (CNFL) **(C)**, corneal nerve branch density **(D)**, beading frequency **(E)**, and bead size **(F)** between control subjects, baseline, and end point among subgroups stratified by the mean HbA1c levels during follow-up. Hatched column; control subjects, open column: at baseline, solid column: mean level **(A)** or at end point **(B–F)**. *p < 0.001 compared with good control subgroup, ^†^*p* < 0.001 compared with fair control subgroup, ^‡^*p* < 0.01 compared with good control subgroup, ^§^*p* < 0.01 compared with baseline, ^||^*p* < 0.001 compared with baseline, ^¶^*p* < 0.01 compared with fair control subgroup, ^#^*p* < 0.05 compared with baseline, **p < 0.05 compared with fair control subgroup.

**Figure 3:**
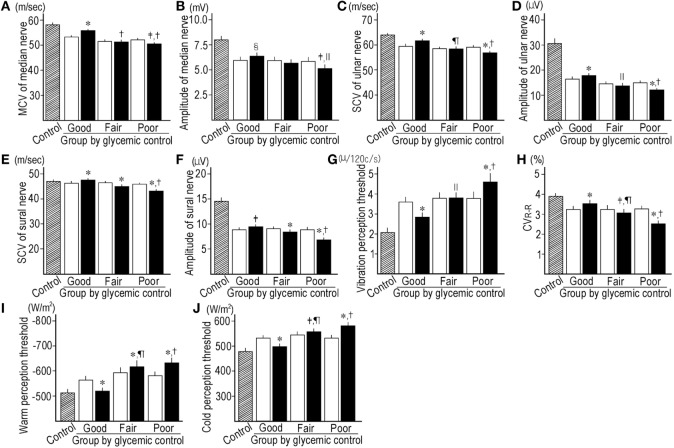
Comparison of motor nerve conduction velocity (MCV) **(A)** and amplitude **(B)** of median nerve, sensory nerve conduction velocity (SCV) **(C)** and amplitude of ulnar nerve **(D)**, SCV **(E)** and amplitude of sural nerve **(F)**, vibration perception threshold **(G)**, CV_R–R_ **(H)**, warm perception threshold (PT) **(I)** and cold PT **(J)** between control subjects (hatched column), baseline (open column), and end point (solid column) in subgroups stratified by the mean HbA1c levels during follow-up. *p < 0.001 compared with baseline, ^†^*p* < 0.001 compared with good control subgroup, ^‡^*p* < 0.01 compared with baseline, ^§^*p* < 0.05 compared with baseline, ^||^*p* < 0.05 compared with good control subgroup, ^¶^*p* < 0.01 compared with good control subgroup.

The HbA1c levels at end point and the mean levels (Figure 2A) were clearly different among subgroups. The decrease in HbA1c levels from baseline in the good GC subgroup was significantly larger than those in other GC subgroups. The retinopathy increased under poor GC. The nephropathy and neuropathy decreased under good GC. Good GC significantly improved all neurophysiological tests, while fair GC worsened some neurophysiological tests. Under poor GC, all neurophysiological functions deteriorated. Many neurophysiological functions under fair and poor GC were inferior to those under good GC (Table 2, Figure 3).

Even under good GC, CNFD (*p* = 0.750) and CNFL (*p* = 0.069) did not improve, while they were significantly higher than those under poor GC (*p* = 0.009 and 0.008, respectively). CNBD, BF, and BS were improved under good GC, and fair GC decreased BS. The poor GC worsened all CNF measures (*p* = <0.001–0.027) (Table 2; Figures 2B–F).

Under fair and poor GC, insulin-providing agents were more frequently prescribed compared with baseline. Insulin-sensitizing agents were more frequently prescribed than at baseline under good and fair GC. Dipeptidyl peptidase-4 (DPP-4) inhibitor was prescribed more frequently than at baseline in all subgroups and was prescribed less frequently under good GC than others (Table 2).

### Associations between Changes in Neuropathy Outcomes and Clinical Factors in All Patients

**Figure 4:**
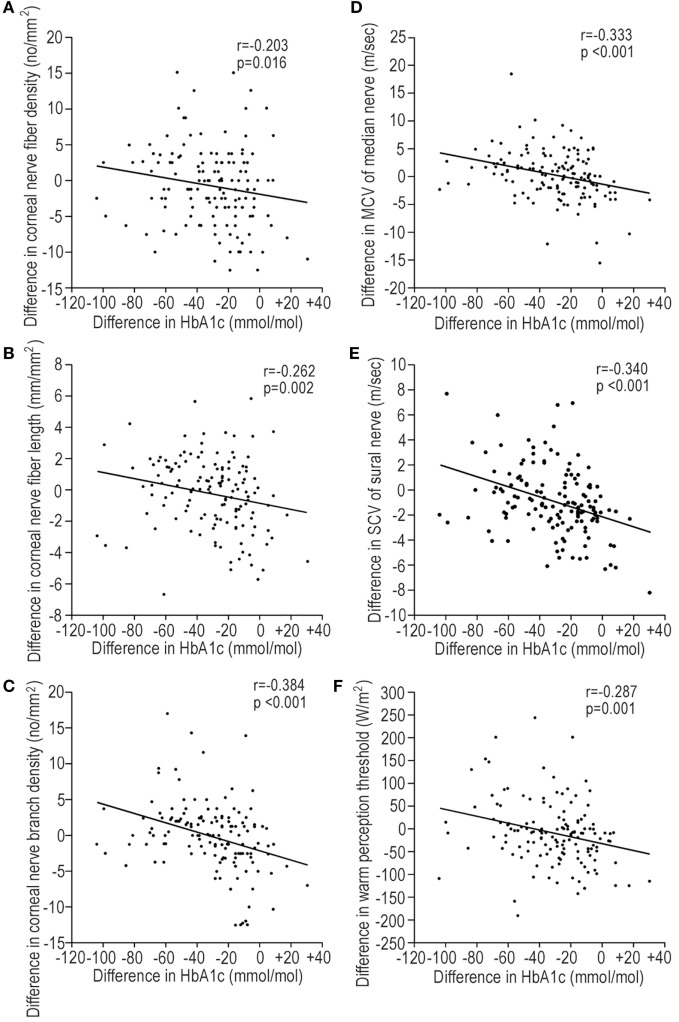
The regression plots representing the correlations between the interval changes in corneal nerve fiber (CNF) measures [**(A)** CNFD, **(B)** CNFL, and **(C)** CNBD] or neurophysiological tests [**(D)** MCV of median nerve, **(E)** SCV of sural nerve, and **(F)** warm perception threshold (PT)] and the interval changes in HbA1c levels during follow-up. Interval changes: value at end point − value at baseline. Abbreviations: CNBD, corneal nerve branch density; CNFD, corneal nerve fiber density; CNFL, corneal nerve fiber length; MCV, motor nerve conduction velocity; PT, perception threshold; SCV, sensory nerve conduction velocity.

The alterations in CNF parameters by GC were closely associated with the mean HbA1c level during follow-up. The duration of diabetes and the HDL-C level were negatively and positively associated with CNBD, respectively, but not other CNF measures. The mean HbA1c level had significant correlations with improvement in neurological dysfunctions in total patients. However, the HbA1c level at baseline did not significantly relate with CNF measures or neurophysiological tests (standard β: −0.139–0.05, *p* = 0.059–0.960). Except for CNFD (standard β: −159, *p* = 0.060) and ulnar nerve amplitude (standard β: −0.142, *p* = 0.098), changes in all other neuropathy outcome measures by GC significantly associated with the magnitude of reduction in HbA1c levels during follow-up (standard β: −0.197–0.368, *p* = <0.001–0.020). The duration of diabetes might worsen the MN MCV. The LDL-C level was positively associated with the VPT. The insulin-providing strategy deteriorated the nerve functions. The DPP-4 inhibitor impaired the SN SCV and amplitude (Table 3). The insulin-sensitizing strategy had no association with nerve functions. The mean SBP and DBP (standard β: −0.005 to −0.159, *p* = 0.962–0.194) had no significant association with interval changes in any neuropathy outcomes. The interval changes in HbA1c levels were correlated with the interval changes in CNF measures and neurophysiological tests (Figure 4).

**Table 3:** Correlation between interval changes by glycemic control in measures of corneal nerve fibers or nerve functions and mean clinical factors during follow-up or hypoglycemic strategies, and the slope of the linear regression of the interval changes in neuropathy outcome measures by 10 mmol/mol HbA1c reduction per year in patients with type 2 diabetes.

Interval changes in neuropathy outcome measures	Mean clinical factors during follow-up period								Hypoglycemic strategy					
	Duration of diabetes mellitus		HbA1c		LDL cholesterol		HDL cholesterol		Insulin providing		DPP-4 inhibitor		Linear regression by 10 mmol/mol annual HbA1c reduction	
							
	Standard β	*p*	Standard β	*p*	Standard β	*p*	Standard β	*p*	Standard β	*p*	Standard β	*p*	Slope	*p*
**Corneal nerve fiber**														

Density	−0.063	0.530	−0.267	**0.023**	−0.069	0.459	0.075	0.379	0.039	0.723	0.035	0.677	0.096	**0.015**
Length	−0.077	0.442	−0.260	**0.025**	−0.036	0.697	0.037	0.661	−0.050	0.648	0.068	0.418	0.049	**0.002**
Branch density	−0.206	**0.023**	−0.242	**0.021**	−0.127	0.128	0.264	**0.001**	−0.076	0.442	−0.131	0.088	0.163	**<0.001**
Beading frequency	−0.147	0.120	−0.316	**0.004**	−0.170	0.052	−0.033	0.679	−0.009	0.932	−0.017	0.828	0.070	**0.002**
Bead size	0.147	0.070	0.399	**<0.001**	−0.006	0.931	−0.063	0.354	0.180	**0.043**	0.151	**0.029**	−0.045	**<0.001**

**Nerve functions**														

MCV of median nerve	−0.240	**0.010**	−0.238	**0.027**	−0.070	0.407	−0.012	0.877	−0.116	0.251	−0.021	0.791	0.136	**<0.001**
Amplitude of ulnar nerve	0.112	0.208	−0.376	**<0.001**	−0.033	0.681	0.102	0.173	−0.290	**0.003**	−0.022	0.774	0.056	**0.007**
SCV of sural nerve	−0.034	0.649	−0.425	**<0.001**	0.068	0.330	0.013	0.835	−0.296	**<0.001**	−0.181	**0.005**	0.101	**<0.001**
Amplitude of sural nerve	−0.095	0.234	−0.371	**<0.001**	−0.109	0.140	0.068	0.310	−0.220	**0.013**	−0.171	**0.012**	0.038	**0.001**
Vibration perception threshold	0.149	0.060	0.398	**<0.001**	0.213	**0.004**	−0.074	0.263	0.175	**0.044**	0.004	0.953	−0.033	**<0.001**
Warm perception threshold	−0.084	0.338	−0.254	**0.014**	0.015	0.857	0.094	0.205	−0.322	**0.001**	−0.003	0.960	1.91	**0.001**
Cold perception threshold	0.101	0.231	0.357	**<0.001**	−0.024	0.760	−0.042	0.556	0.215	**0.022**	0.087	0.227	−2.17	**<0.001**

*Bold p value reveals p < 0.05*.

*DPP-4, dipeptidyl peptidase-4; HDL, high-density lipoprotein; LDL, low-density lipoprotein; MCV, motor nerve conduction velocity; SCV, sensory nerve conduction velocity*.

One important finding in this study is that 10 mmol/mol reduction in HbA1c levels per year significantly improved CNF measures and neurophysiological dysfunctions (Table 3).

In 116 patients without neuropathy at baseline, 14 patients developed neuropathy after follow-up. When the clinical factors at baseline and their mean levels during follow-up were compared between patients with or without the development of neuropathy, the mean HbA1c levels (*p* < 0.001) during follow-up and the duration of diabetes (*p* = 0.004) were the most significant factors. None of patients in good GC subgroup developed neuropathy. Among the neuropathy outcome measures at baseline, the MN MCV (*p* = 0.039) and the SN SCV (*p* < 0.001) in patients developing neuropathy were slower than those in patients without neuropathy.

### The Differential Abilities of CNF Measures and Neurophysiological Tests between Controls and Patients without Neuropathy and between Patients with or without Neuropathy

**Figure 5:**
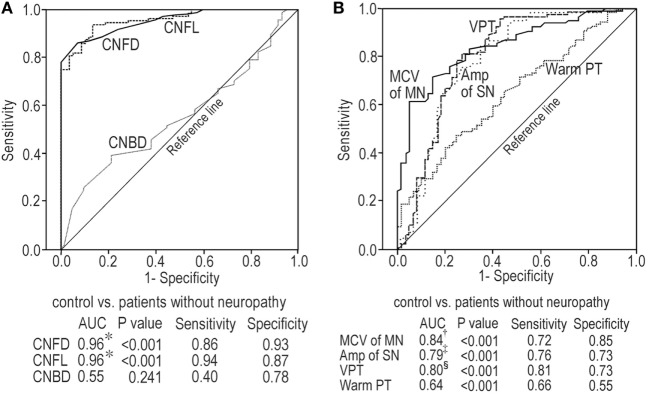
Receiver-operating characteristic curve analyses for corneal nerve fiber (CNF) measures at baseline between control subjects and patients without neuropathy **(A)**; neurophysiological tests at baseline between control subjects and patients without neuropathy **(B)**. *p < 0.001 compared with CNBD, ^†^*p* < 0.001 compared with warm PT, ^‡^*p* < 0.05 compared with warm PT, ^§^*p* < 0.01 compared with warm PT. Abbreviations: Amp, amplitude; AUC, area under curve; CNBD, corneal nerve branch density; CNFD, corneal nerve fiber density; CNFL, corneal nerve fiber length; MCV, motor nerve conduction velocity; MN, median nerve; PT, perception threshold; SN, sural nerve; VPT, vibration perception threshold.

At baseline and end point, CNFD and CNFL had the excellent ability to differentiate between controls and patients without neuropathy and better than CNBD (Table 4; Figure 5A). CNF measures lost differential ability between patients with or without neuropathy. The neurophysiological tests possessed modest differential ability between controls and patients without neuropathy, and the MN MCV, SN amplitude, and VPT were better than warm PT at baseline (Figure 5B). The neurophysiological test still possessed the modest differential ability between patients with or without neuropathy. They had good PPV between controls and patients without neuropathy and good NPV between patients with or without neuropathy. The area under curve of most neuropathy outcome measures at baseline and end point appeared to be similar between controls and patients without neuropathy and between patients with or without neuropathy (Table 4).

**Table 4:** Differential abilities of corneal nerve fiber (CNF) measures and neurophysiological tests between control subjects and diabetic patients without neuropathy, or between diabetic patients with or without neuropathy at the baseline and end point.

		Control subjects vs. diabetic patients without neuropathy at baseline						Diabetic patients with vs. without neuropathy at baseline					
			
		AUC 95%CI	*p-*Value	Sensitivity	Specificity	PPV (%)	NPV (%)	AUC 95% CI	*p*-Value	Sensitivity	Specificity	PPV (%)	NPV (%)
**CNF measures**													

CNF density	Baseline	0.960.93–0.98	**<0.001**	0.86	0.93	96.1	77.8	0.610.49–0.73	0.061	0.60	0.66	27.3	88.4
	End point	0.970.95–0.99	**<0.001**	0.92	0.93	96.4	86.1	0.510.37–0.64	0.916	0.48	0.66	23.1	85.4

CNF length	Baseline	0.960.93–0.98	**<0.001**	0.94	0.87	93.2	88.2	0.620.50–0.74	0.051	0.72	0.59	27.3	90.7
	End point	0.970.95–0.99	**<0.001**	0.93	0.92	95.6	87.3	0.590.47–0.71	0.160	0.52	0.66	24.5	86.4

Corneal nerve branch density	Baseline	0.550.47–0.64	0.241	0.40	0.78	78.0	40.2	0.520.39–0.65	0.806	0.44	0.66	21.6	84.4
	End point	0.590.50–0.67	**0.048**	0.44	0.78	79.7	42.0	0.540.41–0.67	0.525	0.68	0.50	22.7	87.9

**Neurophysiological tests**													

Motor nerve conduction velocity of median nerve (MN)	Baseline	0.840.78–0.90	**<0.001**	0.72	0.85	90.2	60.8	0.650.51–0.78	**0.031**	0.60	0.76	34.9	89.8
	End point	0.820.75–0.88	**<0.001**	0.70	0.85	90.0	59.3	0.580.45–0.71	0.209	0.56	0.63	24.6	86.9

Amplitude of MN	Baseline	0.700.62–0.78	**<0.001**	0.55	0.68	77.1	44.1	0.620.50–0.75	0.059	0.60	0.66	27.8	88.5
	End point	0.730.65–0.80	**<0.001**	0.57	0.78	83.5	48.4	0.550.44–0.67	0.374	0.56	0.60	23.0	86.3

Sensory nerve conduction velocity of sural nerve (SN)	Baseline	0.530.44–0.63	0.511	0.72	0.40	70.0	42.8	1.001.00–1.00	**<0.001**	1.00	0.98	92.7	100
	End point	0.530.45–0.62	0.443	0.54	0.60	72.4	40.4	0.91*0.85–0.96	**<0.001**	0.84	0.83	51.3	96.0

Amplitude of SN	Baseline	0.790.71–0.87	**<0.001**	0.76	0.73	84.6	61.1	0.680.56–0.80	**0.004**	0.56	0.78	35.0	89.1
	End point	0.81*0.74–0.89	**<0.001**	0.81	0.75	86.2	67.1	0.680.56–0.79	**0.004**	0.64	0.66	28.6	89.4

Vibration perception threshold (PT)	Baseline	0.800.72–0.88	**<0.001**	0.81	0.73	85.4	66.6	0.550.42–0.68	0.446	0.60	0.58	23.5	87.0
	End point	0.790.71–0.87	**<0.001**	0.78	0.75	85.7	63.4	0.510.39–0.64	0.829	0.56	0.53	20.3	84.7

Warm PT	Baseline	0.640.56–0.73	**<0.001**	0.66	0.55	73.8	45.2	0.500.38–0.62	0.989	0.52	0.52	18.8	83.3
	End point	0.640.55–0.72	**0.002**	0.53	0.70	77.2	43.3	0.520.40–0.64	0.704	0.64	0.47	20.5	85.7

Cold PT	Baseline	0.650.57–0.74	**<0.001**	0.65	0.57	74.3	45.4	0.530.39–0.66	0.700	0.56	0.63	24.6	86.9
	End point	0.670.59–0.75	<**0.001**	0.67	0.57	75.0	47.2	0.530.40–0.65	0.691	0.56	0.56	21.5	85.5

*p < 0.01 compared with baseline.

*Bold p value reveals p < 0.05*.

*AUC, area under curve; CI, confidence interval; NPV, negative predictive value; PPV, positive predictive value*.

## Discussion

The strict GC is the only strategy for slowing DPN progression in patients with type 2 diabetes. Thus, identifying potentially modifiable risk factors for neuropathy is crucial (23). In type 1 diabetes, cardiovascular risk factors are associated with the neuropathy incidence (23). It is also critical to identify the study cohort with mild to moderate DPN, because severe DPN may be less amenable to intervention (24). The current cohort had mild neuropathy at baseline (NDS: 4.2 ± 0.2). As the SNFs are most likely to show objective responses to interventions (25), SNF measures of DPN are essential to evaluate the efficacy of GC in clinical studies.

The corneal sub-basal nerve plexus is predominantly composed of SNFs (26), and CCM can quantify SNF pathology in diabetic neuropathy (27, 28). A recent longitudinal study in type 1 diabetes without neuropathy revealed that GC, HDL-C, and age impact on CNF measures (29). However, the risk factors associated with the altered CNF measures in type 2 diabetes have never been studied. Because in patients with long-standing type 1 diabetes baseline HbA1c levels predict the DPN development (30), we stratified the patients by the baseline HbA1c levels. HbA1c levels in subgroups decreased to 53.0–56.4 mmol/mol for 4 years. There was no difference in the improvement of neuropathy outcome measures between subgroups. However, the incidence of neuropathy decreased insignificantly in subgroup with the poorest control of HbA1c at baseline. The baseline HbA1c levels were not associated with the interval changes in neuropathy outcomes. The magnitude of reduction in HbA1c levels from baseline had a significant relationship with the changes in many neuropathy outcome measures, but the significance levels were less marked compared with the association with the mean HbA1c levels during follow-up. Then, we divided the patients into three subgroups according to the mean HbA1c level during follow-up. After follow-up, good GC reduced and poor GC increased the incidence of neuropathy. Under good GC, some CNF measures and all neurophysiological functions improved. The significantly larger decrease in HbA1c levels from baseline in good control subgroup due to relatively more inclusion of subgroup-3 members (patients with the highest HbA1c level at baseline) may contribute to the improvement in neuropathy outcomes. The fair control reduced BS but worsened some neurophysiological functions. This might be due to insufficient GC level in this subgroup. Because the mean HbA1c levels during follow-up in fair control subgroup were 55.6 ± 0.2 mmol/mol, this GC level was similar or inferior to those in clinical trials which are not beneficial for the amelioration of DPN (1, 31). Poor GC deteriorated most neuropathy outcome measures. These results indicated that the near-normoglycemia (HbA1c: 47.5 mmol/mol) achieved by GC is beneficial for neuropathy, while the mean HbA1c level higher than 53.0 mmol/mol in fair control subgroup and at end point HbA1c levels in total and subgroups stratified by the baseline HbA1c are not effective to prevent the deterioration of neuropathy outcomes.

In the Kumamoto study (31), the tight GC (HbA1c: 54.1 mmol/mol) prevented NCV but not CAN decline in type 2 diabetes. In the United Kingdom Prospective Diabetes Study (UKPDS) (1), the intensive (HbA1c: 53.0 mmol/mol) and conventional GC (HbA1c: 62.8 mmol/mol) had a similar effect on DPN and CAN. In the ACCORD (Action to Control Cardiovascular Risk in Diabetes) trial (3), the intensive treatment (HbA1c: 45.4 mmol/mol) prevented loss of ankle jerk and light-touch sensation, but resulted in an increased total and CVD-related mortality and severe hypoglycemia. Furthermore, reliable neurophysiological tests were not included. These randomized trials could not establish the optimum GC level for preventing the deterioration of neuropathy outcomes in type 2 diabetes and did not evaluate the SNF morphology.

When patients with recent onset type 1 diabetes were followed up under near-normoglycemia (HbA1c: 47.5 mmol/mol) for 24 years, the decline in nerve functions was completely prevented (32). Although the HbA1c levels of <53.0 mmol/mol is considered to be a reasonable treatment goal (33), more stringent HbA1c goals (≤47.5 mmol/mol) are suggested for selected patients if achievable without hypoglycemia (32). Therefore, the near-normoglycemia is prerequisite for preventing neurophysiological deterioration. These findings were compatible with our study showing that all neurophysiological tests were improved under near-normoglycemia (mean HbA1c: 47.5 mmol/mol).

In patients with type 1 diabetes receiving simultaneous pancreas–kidney transplantation, euglycemia improved CNF measures within 12 months after transplantation (34). In type 1 diabetes, CNFD and CNFL, and BF alter related to the annual mean HbA1c level for previous 7–10 years and 1 year before CCM examination, respectively (17). The CNF measures may improve with different metabolic memory after the establishment of GC. An improved HbA1c level was associated with repair in CNFD but not in CNBD in a small mixed cohort of type 1 and type 2 diabetes (35), indicating the different sensitivities to GC among CNF measures. Because the above study included a small number of type 1 diabetes-dominant patients, their results were different from our study.

As BF and BS showed better association with the mean HbA1c level than other CNF measures, the beads may be more responsible to strict GC than other CNF measures. The beads visualized by CCM are composed of the accumulated mitochondria, glycogen particles, and vesicles (26), and mitochondria play a pivotal role in nerve conduction *via* their adenosine triphosphate production in nerve fibers. We already reported that the hyperglycemia-induced expansion of beads in CNFs might be a predictor of slow NCV in patients with type 2 diabetes (18). However, further investigations are required to evaluate the pathophysiological role of altered beading structure in developing DPN. The near-normoglycemia did not improve CNFD and CNFL, but prevented further declines under persistent hyperglycemia.

As the GC strategy influences DPN (15), we examined the impact of treatment strategies on the changes in neuropathy outcomes by GC. The infrequency of insulin-providing agents and DPP-4 inhibitors in the good GC subgroup than in others may contribute to the tight GC without hypoglycemia. As the insulin-providing strategy was detrimental to most neurophysiological functions, the impact of GC on the neuropathy outcomes should be assessed with careful attention to treatment strategies.

In good GC subgroup, BMI significantly decreased after GC, and hence, good response to lifestyle intervention may improve the neuropathy outcomes (6). Among the components of metabolic syndrome affecting DPN (36), dyslipidemia had a marginal impact on neuropathy outcome measures. Smoking and alcohol consumption, which may influence on DPN (36), had no impact on DPN.

Because the performance and area under curve of most CNF parameters at baseline and end point for differentiating between controls and patients with or without neuropathy were satisfactory, the assessment of neuropathy outcome measures at baseline may predict the subsequent changes especially in those with more severe damage and those that are the subjects that may further benefit from GC. Because CNFD and CNFL excellently differentiated between controls and patients without neuropathy, these are the best measures for detecting the early DPN. Neurophysiological tests had modest sensitivity and specificity. Before and after developing mild neuropathy, high PPV and NPV were found, respectively, suggesting that the neurophysiological tests contribute to the sensitive diagnosis in the early stage and reliable diagnosis after developing neuropathy.

In the current study, with optimal GC, nephropathy decreased, while the retinopathy increased. The initial extreme hyperglycemia paired with rapid and substantial HbA1c reduction might develop the retinopathy (37). The differences from previous studies in the impact of GC on microangiopathy may originate from the differences in patient characteristics (race, age, BMI, duration of diabetes, and HbA1c levels) at baseline (1–3, 31). Perhaps, based on these results, we can control the neuropathy and nephropathy, but not retinopathy. The duration of diabetes in good control subgroup was shorter than that in others. This may contribute to the difference in metabolic memory. Several epidemiological studies (38) suggest that an early intensive GC can reduce the risk of diabetic microangiopathy. The duration of diabetes at baseline was 8.9 years in the current study, and this is an important factor. The emergence of metabolic memory suggests the need for an early aggressive treatment aiming to normalize the metabolic control for minimizing diabetic complications (38).

### Strengths and Limitations

The present study measured the main modifiable risk factors including HbA1c level, blood pressure, and BMI monthly, and the lipid profile, estimated glomerular filtration rate, and ACR every 3 months. Therefore, the mean clinical factors impacting on DPN are representative, and the contribution of HbA1c levels attained by GC and other risk factors on neuropathy outcome measures and other microangiopathies was reliably evaluated. We estimated the morphological and functional measures of SNFs. The altered CNFs were found in all patients with type 2 diabetes at baseline, and near-normoglycemia improved some CNF parameters and all neurophysiological tests. The present study had some limitations. First, we assessed neuropathy outcome measures only at baseline and end point. Ideally, these examinations should be performed annually for assessing the trends of their changes. Second, the development of DPN depends on the metabolic memory (4). Our follow-up period might be too short to reflect the metabolic memory on changes in neuropathy outcome measures. Therefore, we could not detect the impact of baseline HbA1c levels on neuropathy outcomes. Third, this was a retrospective cohort study with a relatively small number of participants. Future prospective studies using a larger cohort are required to consolidate the present results. Finally, the process of selecting CCM images can be more robust to avoid bias. In the current study, based on established protocol and to avoid overlapping of images, we selected at least six high-quality images per subject (39). However, the averaging of parameter values based on multiple CCM images does not necessarily result in good approximations of the respective reference values of the whole image area, indicating the potential for the inevitable local bias when selecting CCM images in the central corneal area (40). Furthermore, cornea is an avascular tissue. In patients with type 2 diabetes, CNFL and CNBD were inversely correlated with tear levels of insulin-like growth factor-binding protein 3 which sequestrates insulin-like growth factor 1 (41). The corneal epithelium-derived neurotrophic factors promote corneal nerve regeneration in mice (42). We acknowledged that there exists a controversy of whether the response of CNF to diabetes or GC is same as that in peripheral sensory nerves in extremities.

In conclusion, the HbA1c level closer to 47.5 mmol/mol achieved mainly with insulin-sensitizing agents and lifestyle modification would be a safe glycemic goal for improving the outcome measures of DPN in patients with poorly controlled type 2 diabetes with mild to moderate neuropathy. The near-normoglycemia is effective for preventing the development of neuropathy and nephropathy, but not retinopathy. However, achieving this is not feasible in many patients.

## Ethics Statement

Written informed consent was obtained from all subjects based on the Declaration of Helsinki. The ethics committee of Ishibashi Clinic approved the protocol of this study.

## Author Contributions

FI designed the study, researched data, and wrote the entire manuscript. MT advised on the statistical analysis, interpreted the results, contributed to writing, and reviewed and revised the whole manuscript. FI and MT are the guarantors of this work and, as such, had full access to all data in the study and take responsibility for the integrity of the data and the accuracy of the data analysis and interpretation.

## Conflict of Interest Statement

The authors declare that the research was conducted in the absence of any commercial or financial relationships that could be construed as a potential conflict of interest.

## Abbreviations

BF, beading frequency; BS, bead size; CCM, corneal confocal microscopy; CNBD, corneal nerve branch density; CNF, corneal nerve fiber; CNFD, corneal nerve fiber density; CNFL, corneal nerve fiber length; DPN, diabetic peripheral neuropathy; ETDRS, Early Treatment Diabetic Retinopathy Study; MCV, motor nerve conduction velocity; NCV, nerve conduction velocity; NDS, neuropathy disability score; SCV, sensory nerve conduction velocity; SNF, small nerve fiber.
